# Supplementary material for: Modulation of Guanylate Cyclase Activating Protein 1 (GCAP1) Dimeric Assembly by Ca2+ or Mg2+: Hints to Understand Protein Activity
Source: Biomolecules. 2020 Oct 5;10(10):1408. doi: 10.3390/biom10101408 (PMC7600425; doi:10.3390/biom10101408)
Supplement: Supplementary file 1 [file biomolecules-10-01408-s001.pdf]

Supplemental data for the paper:

Modulation of Guanylate cyclase activating protein 1 (GCAP1) dimeric assembly by Ca2+ or Mg2+: hints to understand protein activity

By Boni' et al.,

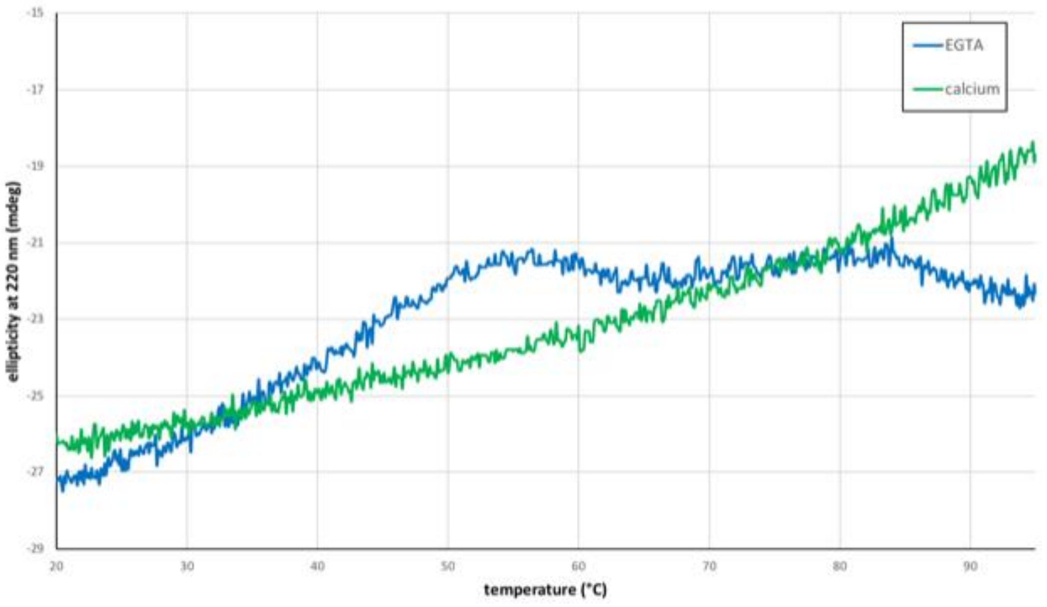

Figure S1. Thermal denaturation of hGCAP1 with (green line) and without Ca^{2+} (blue line) using circular dichroism spectroscopy.

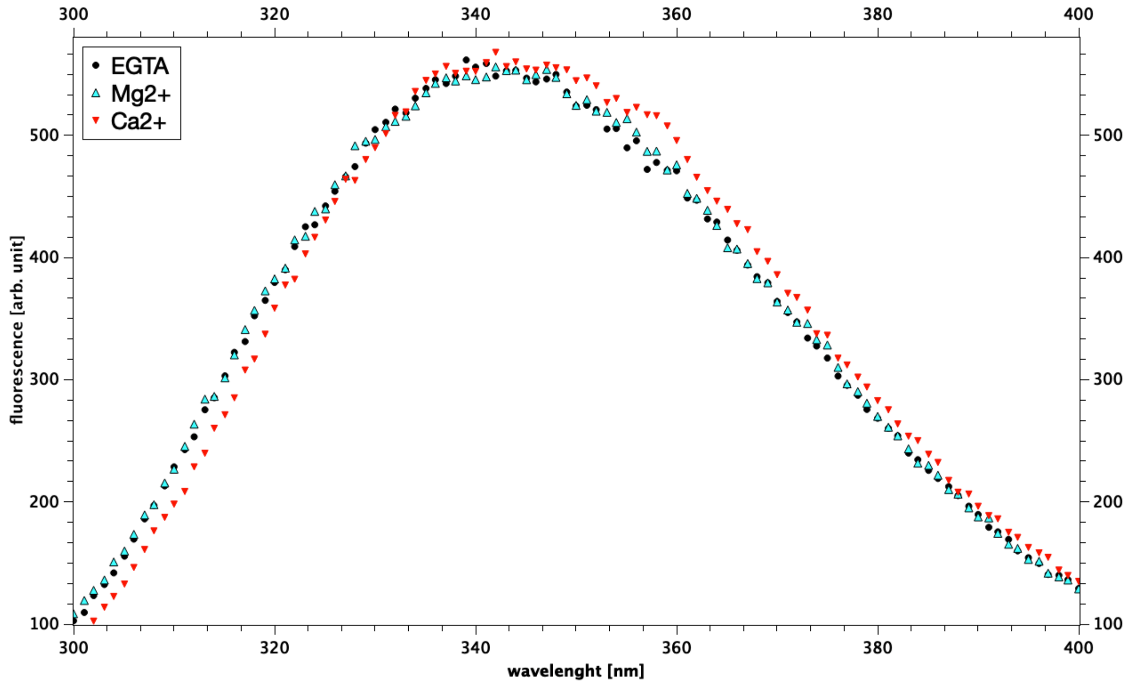

Figure S2. Scaled curves of GCAP1 Trp fluorescence in different conditions: it is possible to note the red shift of the peak corresponding to Ca2+-bound protein.

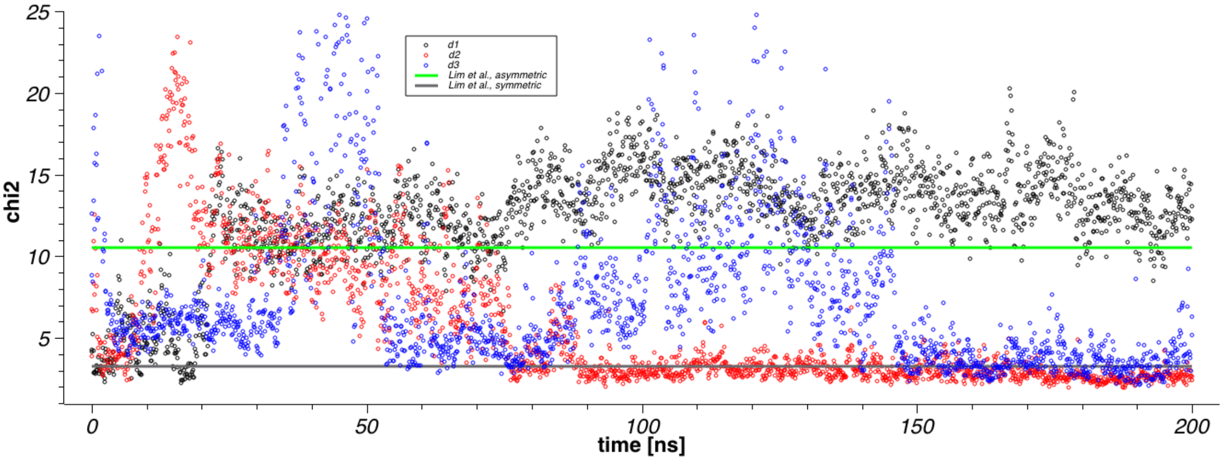

Figure S3. Agreement between SAXS data and dimeric models d1, d2 and d3 without the last 8 residues at the C-ter (calculated using program *Crysol*) along the 200 ns MD simulations. For comparison are reported the chi^2 values of the symmetric and asymmetric models by Lim et al., as gray and green lines, respectively.

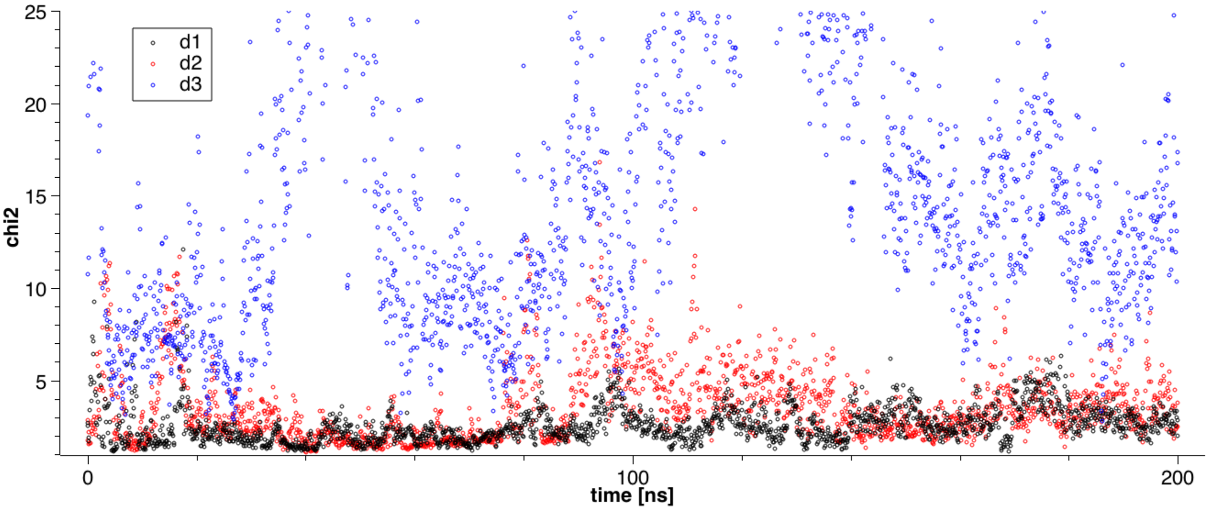

Figure S4. Agreement between SAXS data and dimeric models d1, d2 and d3 (calculated using program *Crysol*) along the 200 ns MD simulation.

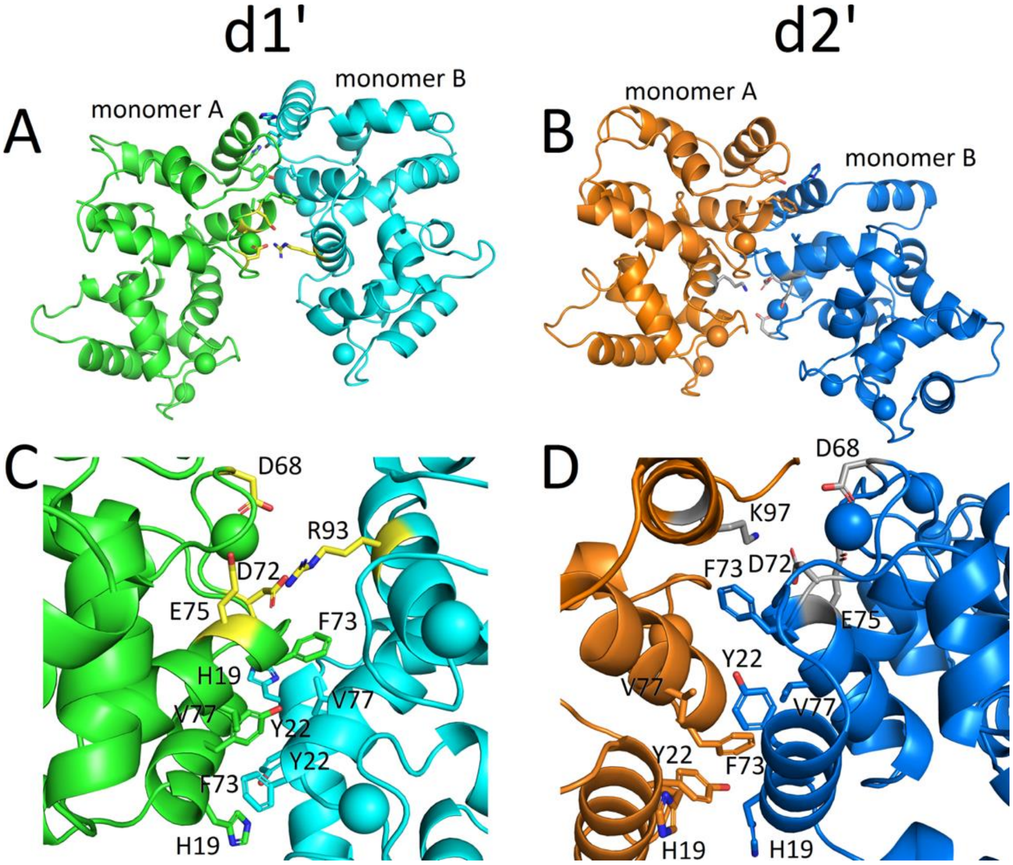

Figure S5. Orientation of monomer B with respect to monomer A in dimers d1′ and d2′ and different pattern of electrostatic interactions. **A)** Three-dimensional representation of model d1′, protein structure is shown in cartoons, monomers A and B are represented in green and cyan, respectively. Ca^{2+} ions are shown as spheres and colored according to each monomer, residues H19, Y22, F73 and V77 are depicted as sticks and colored according to each monomer, residues R93, D68, D72 and E75 are represented as yellow sticks, O and N atoms of the sidechains are colored in red and blue, respectively. **B)** Three-dimensional representation of model d2′, protein structure is shown in cartoons, monomers A and B are represented in orange and cyan, respectively. Ca^{2+} ions are shown as spheres and colored according to each monomer, residues H19, Y22, F73 and V77 are depicted as sticks and colored according to each monomer, residues R93, D68, D72 and E75 are represented as grey sticks, O and N atoms of the sidechains are colored in red and blue, respectively. **C)** Closer view of the interface of dimer d1′ and **D)** d2′, protein structure is depicted as in panels A and B, respectively.
